# Recurrent ventricular fibrillation treated with scar homogenization in a patient with arrhythmogenic cardiomyopathy

**DOI:** 10.1016/j.hrcr.2024.01.003

**Published:** 2024-01-19

**Authors:** Jae-Sun Uhm, Su Kyung Oh, Je-Wook Park, Boyoung Joung, Hui-Nam Pak, Moon-Hyoung Lee

**Affiliations:** ∗ Division of Cardiology, Department of Internal Medicine, Yongin Severance Hospital, Yonsei University College of Medicine, Yongin-si, Republic of Korea; † Biosense Webster, Johnson & Johnson, Inc, Seoul, Republic of Korea; ‡ Division of Cardiology, Department of Internal Medicine, Severance Hospital, Yonsei University College of Medicine, Seoul, Republic of Korea

**Keywords:** Arrhythmogenic cardiomyopathy, Arrhythmogenic right ventricular cardiomyopathy, Catheter ablation, Scar homogenization, Ventricular fibrillation, Ventricular tachycardia

**Key Teaching Points**

• Fast monomorphic ventricular tachycardia (VT) may trigger ventricular fibrillation (VF).
• Scar homogenization could reduce the VT burden, which may result in a reduction in the VF burden.
• Scar homogenization may be effective in reducing VF burden in patients with arrhythmogenic cardiomyopathy.

## Introduction

Arrhythmogenic cardiomyopathy is a myocardial disease characterized by the loss of myocytes and fibrofatty replacement of the ventricular myocardium, predisposing to ventricular tachycardia (VT) or fibrillation (VF), and sudden cardiac arrest.^1^ An implantable cardioverter-defibrillator (ICD) is the only effective therapy for the prevention of sudden cardiac death.

Scar homogenization is a catheter ablation strategy for VT in patients with cardiomyopathy. Potential VT circuits in the scar area can be eliminated via scar homogenization.^2^ However, the effects of scar homogenization on VF remain unknown. Here, we report a case of recurrent VF in a patient with arrhythmogenic cardiomyopathy who was treated with scar homogenization.

## Case report

**Figure 1:**
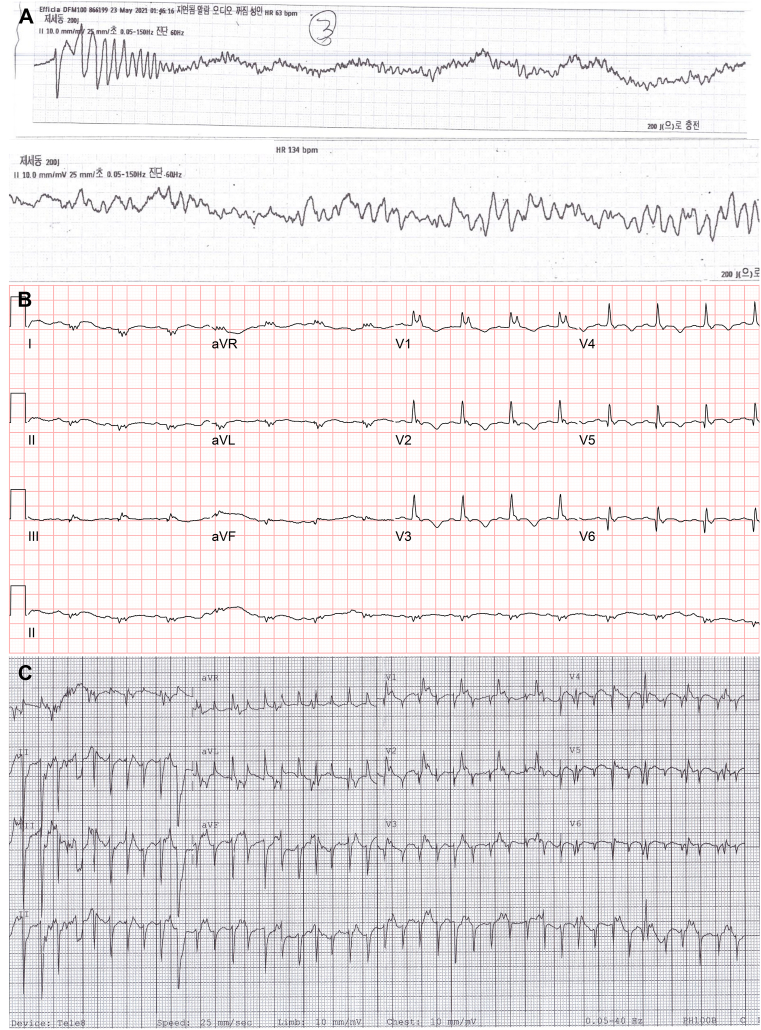
**A:** Rhythm strip during sudden cardiac arrest shows polymorphic ventricular tachycardia (VT) and ventricular fibrillation. **B:** After defibrillation, electrocardiography (ECG) shows normal sinus rhythm, low voltage, atypical right bundle branch block, T-wave inversion, and QT prolongation. **C:** ECG performed during dyspnea and chest discomfort shows parasystole including sinus tachycardia and VT.

**Figure 2:**
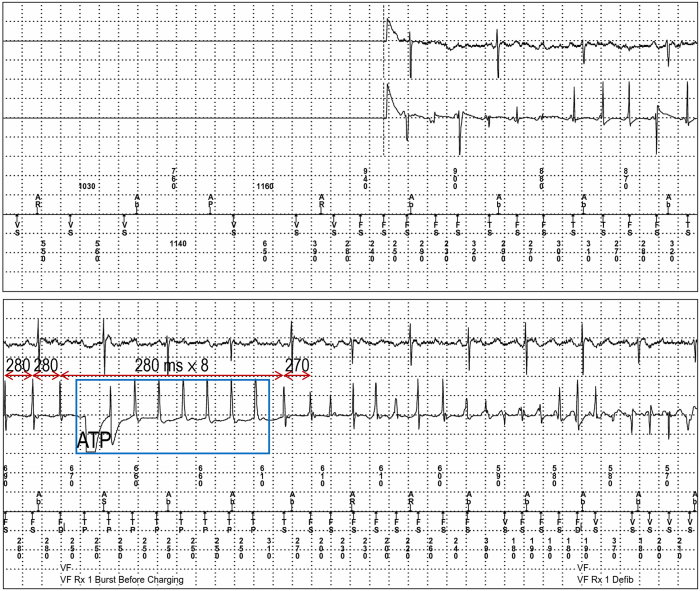
Implantable cardioverter-defibrillator electrograms reveal that the ventricular tachyarrhythmia initiated in a form of polymorphic ventricular tachycardia (VT) and monomorphic VT degenerated into ventricular fibrillation. ATP = antitachycardia pacing.

**Figure 3:**
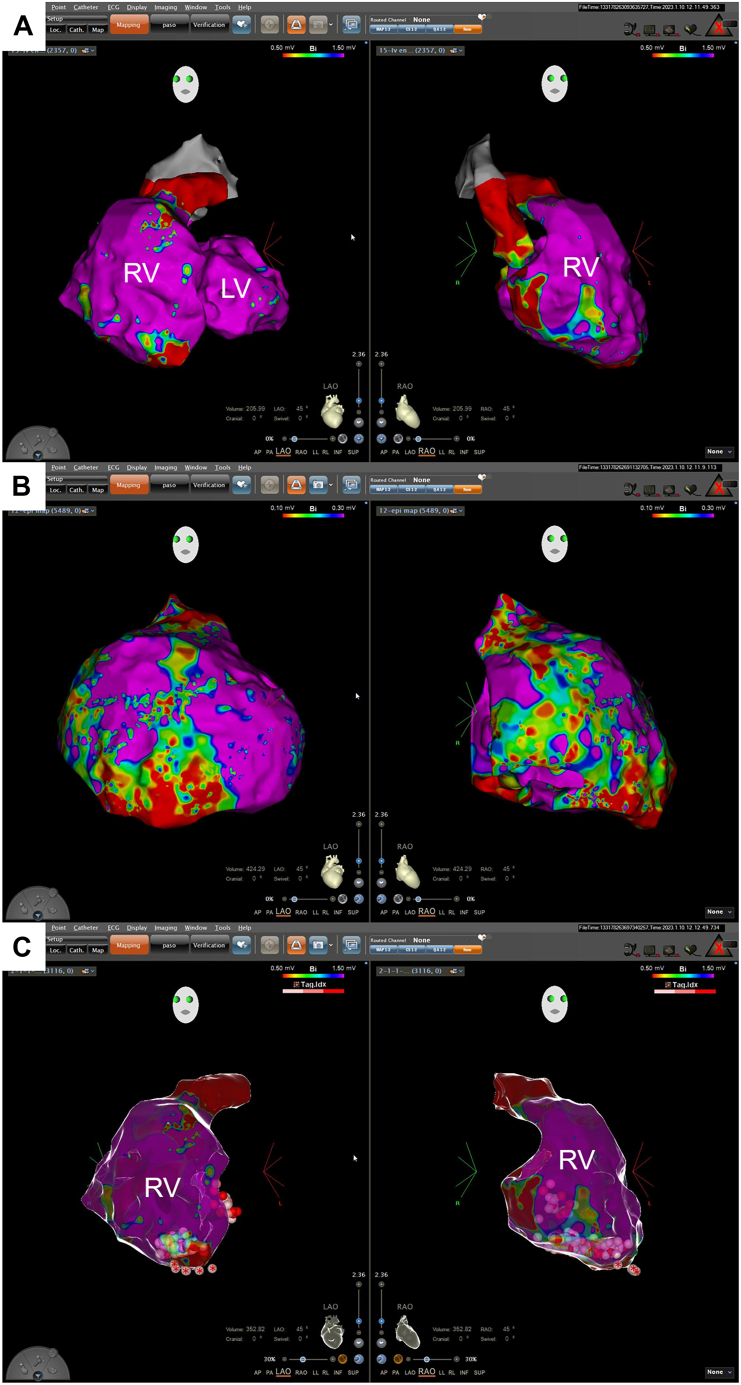
**A, B:** Three-dimensional endocardial (**A**) and epicardial (**B**) voltage map reveals low-voltage (<0.5 mV at the endocardium and <0.1 mV at the epicardium) scar zone at the right ventricle (RV) apex. **C:** Scar homogenization at the endocardial and epicardial RV apex was performed. LV = left ventricle. ∗ indicates epicardial ablation point.

A 28-year-old man visited the emergency room because of sudden-onset dyspnea and chest discomfort. At the entrance to the emergency room, the patient lost consciousness and collapsed. An electrocardiogram (ECG) revealed polymorphic VT and VF at that time (Figure 1A). Urgent defibrillation was performed and VF was terminated. After defibrillation, ECG revealed sinus rhythm, low voltage, atypical right bundle branch block, T-wave inversion, and QT prolongation (Figure 1B). The deflection at the end of the QRS complex in leads V_1_–V_3_ could be epsilon wave. Echocardiography revealed akinesia of the right ventricular (RV) free wall, hypokinesia of the global left ventricular (LV) wall, 20% LV ejection fraction (LVEF), and 15% RV fractional area change. These findings are consistent with those of arrhythmogenic cardiomyopathy. Coronary angiography revealed no evidence of coronary artery disease. Cardiac magnetic resonance imaging revealed a 22% LVEF and a 6% RV ejection fraction. However, delayed enhancement of the myocardium could not be evaluated because of poor image quality. During hospitalization, the patient complained of sudden-onset dyspnea and chest discomfort. At that time, ECG monitoring revealed parasystole, including monomorphic VT and sinus tachycardia. VT has a heart rate of 250 beats/min and probable origin of the RV apex. Sinus tachycardia has a heart rate of 120 beats/min and atypical right bundle branch block morphology (Figure 1C). This ECG suggests that the myocardium involved in the VT might be electrically isolated from the other parts of the ventricle. VT spontaneously terminated. During the tachycardia, the patient felt presyncope and lay calm on his hospital bed. His radial pulse was very fast and weak. The patient reported that he experienced the same symptoms as those to the aforementioned presentation at the emergency room. A dual-chamber ICD was implanted for secondary prevention. Sacubitril/valsartan 200 mg twice a day, bisoprolol 5 mg twice a day, furosemide 40 mg once daily, spironolactone 25 mg once daily, dapagliflozin 10 mg once daily, ivabradine 7.5 mg twice a day, and amiodarone 200 mg once daily were administered for the management of heart failure and ventricular tachyarrhythmia. The patient has been uneventful for 10 months, but experienced dyspnea, chest discomfort, and ICD shock 10 months after ICD implantation. The ICD electrograms revealed that 5 and 3 ICD shocks were delivered because of VF and atrial fibrillation with a rapid ventricular response, respectively. To reduce the VF burden, a left cervical stellate ganglion blockade was performed^3^ and amiodarone 400 mg once daily was administered. However, within a month, 3 instances of ICD shocks for VF were delivered. The ICD electrograms revealed that the ventricular tachyarrhythmia initiated in a form of polymorphic VT and monomorphic VT degenerated into VF (Figure 2). Antitachycardia pacing was performed during VT but failed to terminate VT. We decided to perform an electrophysiological study and catheter ablation for recurrent VF using a 3-dimensional electroanatomical mapping system (CARTO; Johnson & Johnson, Diamond Bar, CA) under general anesthesia. In the electrophysiology lab, a DecaNav catheter (Johnson & Johnson) and His-RV catheter (Japan Lifeline, Tokyo, Japan) were placed in the coronary sinus and RV apex, respectively. VF was induced by programmed electrical stimulation of the RV apex. A percutaneous pericardial puncture was performed using the subxiphoid approach. Voltage mapping of the endocardial and epicardial RV and LV was performed with a PentaRay catheter (Johnson & Johnson) and DecaNav catheter during atrial pacing. The bipolar voltage map revealed a low-voltage zone at the endocardial and epicardial RV apex, RV free wall, and RV outflow tract (Figure 3A and 3B). We considered the low-voltage zone as the myocardial scar and performed scar homogenization at the endocardial and epicardial RV apex because ECG revealed that VT could originate from the RV apex (Figure 3C). In addition, point-by-point catheter ablation targeting Purkinje potentials at the LV-side septum was performed because we had to eliminate any possibility of ventricular arrhythmia for this critically ill patient. Because various morphologies of premature ventricular complexes (PVCs) rarely appear during the procedure, catheter ablation for PVC could not be performed. For 18 months after catheter ablation for VF, he had no symptoms, and neither VT nor VF had developed. LVEF mildly improved and RV systolic function was stationary (37% of LVEF and 17% of RV fractional area change by echocardiography) after 18 months of heart failure management, including medical therapy and cardiac rehabilitation.

## Discussion

The present case suggests that scar homogenization may be effective in reducing VF burden. Herein, we speculate that VF followed monomorphic VT originating from the scar area at the RV apex, judging from ECG monitoring and ICD electrograms. Monomorphic VT may trigger VF. Scar homogenization could reduce the VT burden, which may result in a reduction in the VF burden. Although catheter ablation for the Purkinje potentials was performed, it might not be effective. This was because the PVC originating from the Purkinje system was not a trigger for VF and VF episodes were initiated from monomorphic VT. VT was initiated without PVC (Figures 1 and 2, and Supplemental Figures 1 and 2). Although the exact reason why the QRS amplitude of VT is initially low and then increases is unclear, the VT might begin from small reentrant circuit and propagate to large reentrant circuit.

The mechanisms underlying VF are not fully understood. Four stages of VF development and progression have been suggested: initiation, transition, maintenance, and evolution.^4^ VF is usually initiated by PVCs or degeneration of the fast VT.^5^ PVC-triggered VF generally originates from the ventricular outflow tract,^6^ Purkinje system,^7^ and papillary muscles.^8^ As a mechanism of transition from PVCs to VF, PVCs may produce wavefronts, which propagate through the myocardium, leading to functional reentry.^9^ Multiple unstable circulating wavefronts may maintain VF.^10^ In the evolution stage, persistent VF results in myocardial ischemia and electrical remodeling, which leads to the sustainability of VF. The most widely used catheter ablation strategy for VF is ablation of PVC-triggered VF.^6^^,^^11^ However, in patients with infrequent PVCs of multiple origins, catheter ablation is challenging. As the Purkinje system may play a critical role in the initiation stage, catheter ablation for Purkinje potentials can be a cornerstone of catheter ablation for VF in patients with inherited primary arrhythmia syndromes and ischemic or nonischemic cardiomyopathies.^6^^,^^11^ However, catheter ablation for VF is challenging and its outcomes are not always satisfactory.

An endocardial and epicardial substrate mapping and ablation strategy is effective in VT treatment in patients with arrhythmogenic cardiomyopathy.^12^ Target abnormal electrograms are characterized by low-voltage (<0.5 mV), longer (>80 ms), and more fractionated (≥5 deflections) electrograms. Myocardial scar is usually formed within the subepicardial and midmyocardial region at RV inferior wall, angle, and anterior wall.^12^ In Brugada syndrome, target abnormal electrograms are defined as low-voltage (≤1 mV), longer (>80 ms), late (beyond QRS complex), fractionated (≥2 deflections) electrograms. Abnormal electrograms are common in the epicardial RV outflow tract.^13^ How to perform catheter ablation for VF in patients with arrhythmogenic cardiomyopathy has not yet been established. We obtained favorable results with scar homogenization of the RV apex in a patient with arrhythmogenic cardiomyopathy who experienced recurrent VF. Further studies are needed to verify the effects of scar homogenization on VF. This finding should not be generalized to patients without cardiomyopathy because the myocardial scarring may not play a critical role in the initiation and maintenance of VF in these patients.

## Conclusion

The catheter ablation strategy for scar homogenization may be effective in reducing VF burden in patients with arrhythmogenic cardiomyopathy.

## Disclosures

All of the authors declare no conflict of interest.
