# Supplementary material for: Recurrent ventricular fibrillation treated with scar homogenization in a patient with arrhythmogenic cardiomyopathy
Source: HeartRhythm Case Rep. 2024 Jan 19;10(4):250–4. doi: 10.1016/j.hrcr.2024.01.003 (PMC11096426; doi:10.1016/j.hrcr.2024.01.003)
Supplement: Supplementary Figures 1 and 2 [file mmc1.docx]

**
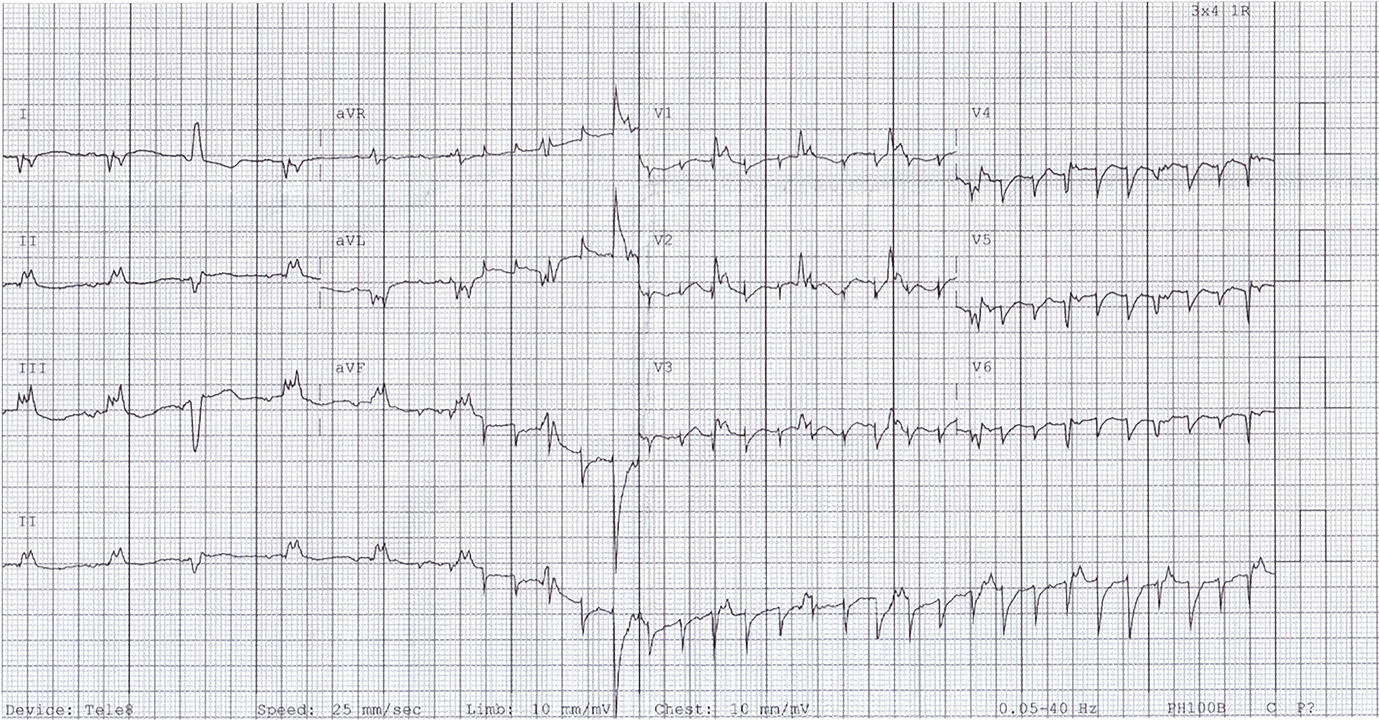
**

**Supplementary figure 1.** ECG at the onset of VT. VT, ventricular tachycardia


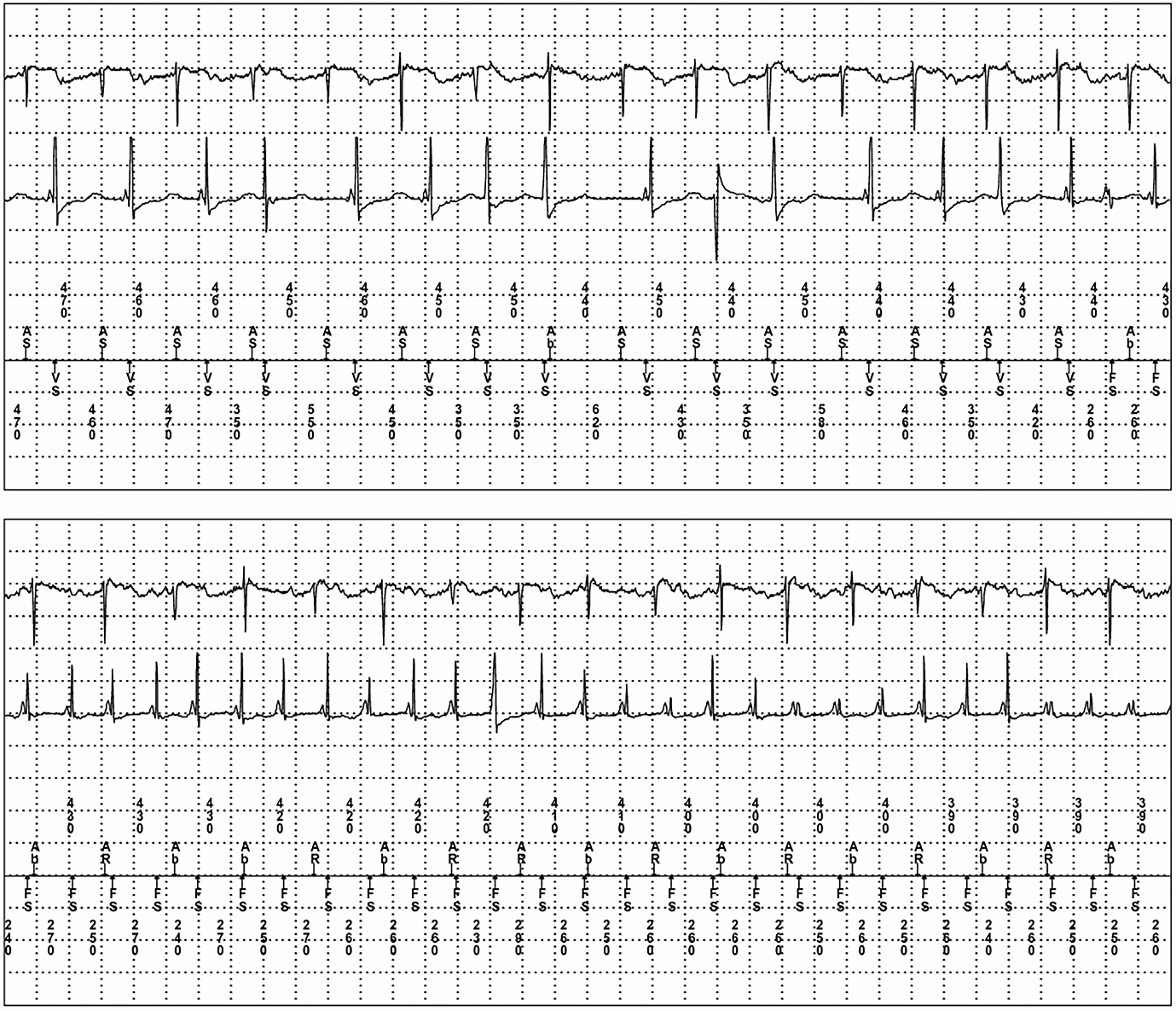


**Supplementary figure 2.** ICD electrogram at the onset of VT. ICD, implantable cardioverter-defibrillator; VT, ventricular tachycardia
